# Food Waste–Derived Organic Fertilizers: Critical Insights, Agronomic Impacts, and Pathways for Sustainable Adoption

**DOI:** 10.1155/ijfo/1551054

**Published:** 2025-11-11

**Authors:** Md. Suhel Mia, Wahidu Zzaman

**Affiliations:** ^1^ Department of Food Engineering and Tea Technology, Shahjalal University of Science and Technology, Sylhet, Bangladesh, sust.edu

**Keywords:** compost, digestate, food waste, pyrolysis, soil health, valorization, vermicompost

## Abstract

Food waste is one of the fastest growing sustainability challenges, wasting scarce resources and aggravating environmental degradation. Its valorization into organic fertilizers provides a critical opportunity to recover nutrients, reduce landfill burdens, and strengthen circular bioeconomy strategies. This review critically examines FW‐derived organic fertilizers (FWOFs) across four major conversion routes including composting, vermicomposting, anaerobic digestion, and pyrolysis and also evaluates their impacts on soil health, nutrient cycling, crop yield and quality, and environmental trade‐offs. We emphasize that while FWOFs offer multiple benefits, outcomes remain highly variable due to heterogeneity in feedstocks, processing methods, and application practices. Evidence highlights the strong potential for improving soil organic matter, water retention, and micronutrient supply but also raises unresolved risks from heavy metals, microplastics, and the survival of pathogens. By integrating multiscale evidence, this review provides a fertilizer‐focused perspective that identifies critical knowledge gaps, standardization needs, and adoption pathways. In conclusion, this work underscores both the opportunities and limitations of FWOFs, offering concise guidance for advancing sustainable agriculture and circular bioeconomy practices.

## 1. Introduction

Food waste refers to the loss of food quality or quantity caused by actions and choices made by food retailers, service providers, and end users [1]. It has emerged as one of the most pressing sustainability challenges, affecting every stage of the agri–food chain from farm to table. Global population growth and rising food demand have intensified this issue, generating unprecedented volumes of food and agricultural residues [2, 3]. The consequences are multifaceted: In addition to the direct economic losses from wasted inputs such as labor, infrastructure, and energy, food waste accelerates environmental degradation. Within agriculture, poor waste management is linked to deforestation, soil depletion, and land degradation. Valuable resources like arable land, fresh water, and fossil energy are squandered when food is discarded rather than consumed [4]. If mismanaged, food waste also disrupts ecosystems, driving biodiversity loss through unsustainable land conversion and input‐intensive farming systems aimed at compensating for waste‐driven deficits. Globally, food waste contributes an estimated 8%–10% of anthropogenic greenhouse gas emissions [5]. Its disposal is particularly problematic: Landfilled food waste decomposes anaerobically to release methane, while incineration produces significant CO₂ emissions [6]. Moreover, the entire life cycle of food production—from cultivation and harvesting to transport and storage—intensifies the climate burden by consuming vast amounts of energy and other inputs. Additional nitrous oxide emissions from fertilizer and pesticide use further magnify the sector’s impact. Hence, sustainable management of food waste is essential to conserve resources, mitigate climate forcing, and support long‐term environmental resilience [7, 8]. Confronting food waste is integral to both climate mitigation and sustainable resource use. The United Nations Sustainable Development Goal 12.3, which is aimed at halving food loss and waste by 2030, underscores the urgency of developing effective and scalable solutions. Solutions that divert food waste from landfills and transform it into value‐added products can significantly reduce methane emissions from anaerobic decomposition and carbon dioxide from incineration, while advancing sustainability.

**Figure 1:**
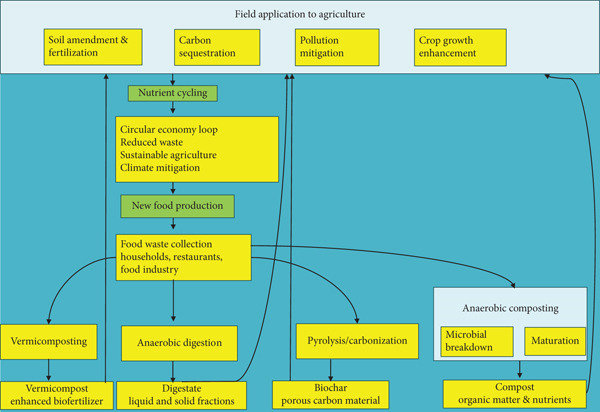
Circular economy flows from food waste to field application.

Among many strategies, valorization through technologies that transform food waste into organic fertilizers stands out for its alignment with circular economy principles. By converting residual food into nutrient‐rich materials, these pathways reduce environmental damage and extend the utility of biological resources, thereby enhancing both ecological and economic performance (Figure 1). For instance, large‐scale composting programs in South Korea and Japan have successfully diverted food waste to produce standardized organic fertilizers, reducing landfill dependence and improving urban soil quality [9, 10]. Similarly, anaerobic digestion (AD) initiatives convert food waste into both renewable energy and digestate fertilizers, demonstrating the dual benefits of energy recovery and nutrient recycling [11, 12]. In many low‐ and middle‐income countries, community‐level vermicomposting projects using vegetable market residues have shown measurable improvements in soil fertility and crop yields, highlighting practical applicability across diverse contexts [13, 14].

Unlike many previous reviews that primarily catalog food waste valorization options, this work narrows the focus to organic fertilizer pathways, critically comparing technologies through the lenses of nutrient recovery, soil–plant impacts, contamination risks, economic viability, and policy frameworks. It moves beyond descriptive lists by integrating multiscale evidence (lab, field, and meta‐analyses) and highlighting systemic gaps in standardization, regulation, and adoption. This critical, fertilizer‐first perspective allows for deeper synthesis of disparate findings and identification of research gaps. The innovation of this review lies in presenting a fertilizer‐centered perspective that synthesizes fragmented evidence into a structured evaluation, offering a more decision‐oriented framework than earlier descriptive overviews. This makes the work particularly relevant for both scientific advancement and practical application in sustainable agriculture.

## 2. Food Waste as a Resource for Organic Fertilizer

### 2.1. Sources and Composition of Food Waste

**Figure 2:**
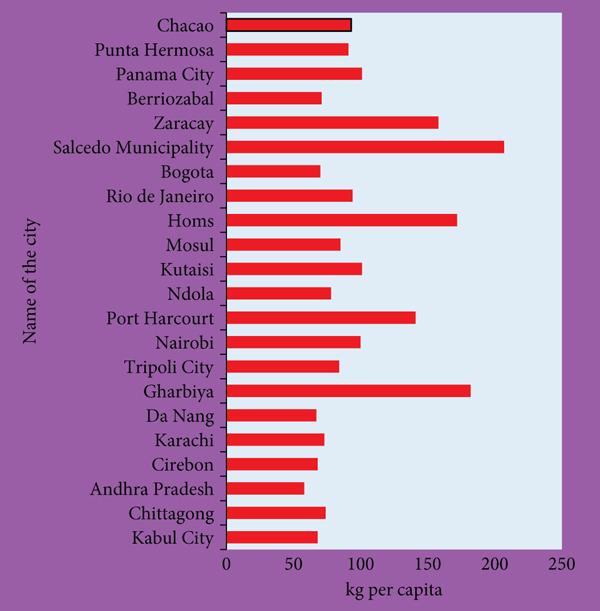
Household food waste in different cities [15].

Food waste is a heterogeneous mixture of discarded raw materials, processing by‐products, and postconsumer residues that vary widely in nutritional quality and safety. For fertilizer production, this heterogeneity is both a strength (provides a reservoir of organic matter and plant nutrients) and a limitation (complicates standardization and quality control). Globally, the main contributors to food waste are agroindustrial processors, food service establishments, and retail chains. Also, another primary source of food waste generation in the globe is household food waste (Figure 2). Each generates waste with distinct nutrient profiles [16]. For example, fruit and vegetable residues are typically high in moisture and rich in potassium (K) and micronutrients, whereas cereal and bakery waste tends to provide higher carbohydrate content but lower mineral availability. Animal‐derived wastes such as meat scraps or dairy by‐products may contribute nitrogen (N) and phosphorus (P) but simultaneously pose higher risks of pathogenic contamination, requiring additional treatment before agricultural application [17, 18].

The nutrient composition of food waste directly influences its suitability as an organic fertilizer. N, P, and K remain the primary macronutrients of interest, but their concentrations are inconsistent. Studies report that fruit peel wastes such as banana or citrus contain appreciable amounts of K, while vegetable trimmings contribute calcium and magnesium. On the other hand, cereal‐based waste generally provides lower NPK values and needs cocomposting with N‐rich substrates such as manure to achieve a balanced C/N ratio. This imbalance is critical because a high carbon‐to‐N ratio can slow down decomposition and reduce fertilizer efficiency [19]. A critical challenge, therefore, is ensuring nutrient uniformity across batches, which is seldom addressed in descriptive studies but is central to real‐world scalability.

Another dimension of food waste composition is contamination with undesirable elements. Household and municipal sources often contain plastic fragments, glass, or heavy metals leached from packaging, while agroindustrial waste is comparatively “cleaner” but may still harbor pesticide residues or mycotoxins. If these contaminants persist in the fertilizer product, they may compromise soil quality, plant health, and ultimately food safety. Regulatory frameworks in the EU and the United States have established thresholds for heavy metals like cadmium (Cd) and lead (Pb) in organic amendments [20]. Thus, a more critical perspective requires not only quantifying beneficial nutrients but also assessing toxicological risks. Moisture content is another key parameter affecting the valorization potential of FW. High‐moisture wastes such as fruit pulp or vegetable trimmings decompose rapidly, enhancing microbial activity during composting but also raising logistical challenges for transport and storage. Dry wastes like rice husks or bakery products are easier to handle but often need moisture adjustment for efficient microbial degradation. A balanced feedstock mixture, blending wet and dry fractions, has been proposed as a practical solution, though such optimization is rarely practiced at scale due to inconsistent waste supply chains [21, 22].

Food waste offers a nutrient‐rich substrate for organic fertilizer production, but its compositional variability, contamination risks, and C/N imbalances present substantial challenges. A critical takeaway is that future research should move beyond cataloging nutrient levels and instead focus on standardizing feedstock classification, developing pretreatment protocols to mitigate contaminants, and designing cocomposting strategies that optimize nutrient availability. Such approaches will bridge the gap between laboratory‐scale demonstrations and industrial‐scale applications, making FWOFs a credible alternative to synthetic inputs.

### 2.2. Nutrient Potential

The value of food waste as a fertilizer source lies in its nutrient composition, particularly its macronutrient and micronutrient content. A critical analysis reveals that food waste offers significant potential to substitute or complement synthetic fertilizers, but only if challenges related to nutrient stability, consistency, and environmental safety are properly managed [23]. N is the most limiting nutrient for crop production worldwide, and food waste streams such as dairy residues, meat by‐products, and vegetable trimmings can supply substantial amounts of organic N. Most food waste contains N primarily in organic compounds, which require microbial mineralization before being plant available. This dependency introduces uncertainties in synchronization between nutrient release and crop demand, often overlooked in fertilizer potential studies. Similarly, P from FW, commonly present in bones, seeds, and dairy residues, is essential but may be bound in organic or insoluble forms, limiting its bioavailability. Thermal or biological pretreatments such as AD and composting can increase P solubility, but they also entail additional costs and operational complexity. K, abundant in fruit and vegetable peels, is generally more available and less problematic, though leaching losses during storage or composting can diminish its recovery efficiency.

Food waste also contributes secondary nutrients (Ca, Mg, and S) and trace elements (Fe, zinc [Zn], Mn, Cu, and B) that are often absent in synthetic fertilizers. For example, citrus peels are rich in calcium and magnesium, while banana peels provide both K and manganese [24]. These micronutrients enhance soil fertility and plant health, supporting FW’s role as a more holistic soil amendment. However, the micronutrient composition varies widely with feedstock type and regional dietary patterns. Critical evaluation suggests that without consistent compositional profiling, relying on FWOFs as a stable micronutrient source remains speculative.

Several studies compare the nutrient content of FW‐based composts with conventional synthetic fertilizers. While food waste products usually provide lower nutrient concentrations, they offer additional organic matter that improves soil structure, microbial diversity, and water retention—benefits not captured in simple NPK comparisons [25, 26]. However, the slower nutrient release rate of FWOFs can be both a strength (reducing nutrient leaching) and a limitation (potential mismatch with crop nutrient demand). Thus, the nutrient potential should not only be expressed as total content but also assessed in terms of release dynamics, bioavailability, and agronomic efficiency. Another challenge is nutrient imbalance. Many food waste streams have high K but insufficient N or P, necessitating cocomposting with manure or other nutrient‐rich wastes to achieve an optimal fertilizer formulation. Yet, this requirement increases process complexity and may reduce cost competitiveness compared to chemical fertilizers.

### 2.3. Contamination Risks

While food waste offers substantial nutrient potential, its use as a fertilizer is constrained by contamination and safety risks. These include heavy metals, pathogens, persistent organic pollutants, antibiotic residues, and emerging contaminants such as microplastics. Failure to address these issues undermines both the agronomic effectiveness and public acceptance of FWOFs. Municipal solid waste and food‐derived composts often exceed background soil levels of Cd, Pb, and Zn, raising long‐term concerns for soil accumulation and food chain transfer. Cd is particularly critical because of its mobility and phytotoxicity. The new EU Fertilising Products Regulation (2019/1009) introduced stringent limits for heavy metals in fertilizers, with Cd thresholds as low as 20 mg/kg P₂O₅ [27]. This indicates increasing regulatory scrutiny, but implementation in low‐ and middle‐income countries remains limited. Importantly, while pretreatment and source separation can reduce contamination, variability across waste streams makes consistent compliance challenging.

Untreated food waste carries high microbial loads, including *Salmonella*, *Escherichia coli*, and *Listeria*. If composting or AD conditions are inadequate, pathogen survival and regrowth are possible. Regulations such as the US EPA’s Processes to Further Reduce Pathogens (PFRP) mandate maintaining compost temperatures at ≥ 55°C for 3–15 days (depending on the method) to achieve pathogen kill [28]. Yet, field‐scale operations often fail to achieve these standards consistently, especially in informal recycling systems. This raises concerns not only for direct soil contamination but also for the occupational health of workers handling food waste compost.

Microplastics, originating from packaging residues and incomplete depackaging, are increasingly detected in composts and digestates. A recent review emphasized that microplastic particles persist in soil, alter microbial activity, and can act as carriers for heavy metals and organic pollutants. The UK PAS 100:2018 compost quality standard, therefore, limits plastic contamination to ≤ 0.12% (m/m) [29]. However, micro‐ and nanoplastic quantification remains methodologically inconsistent, complicating risk assessments. Beyond plastics, residues of pharmaceuticals and pesticides have also been reported in FW‐based fertilizers, raising concerns about long‐term ecological and human health implications.

Animal‐derived FW, including meat scraps and dairy residues, may carry antibiotic residues and resistant bacteria. Composting can reduce microbial loads, but not necessarily resistance genes. Thus, food waste valorization into fertilizers could inadvertently contribute to antimicrobial resistance dissemination in agroecosystems. This aspect remains underexplored and should be prioritized in risk assessment frameworks [30]. Food waste offers a renewable source of plant nutrients, but it is not risk‐free. Heavy metals, pathogens, microplastics, and antibiotic residues represent critical barriers that must be addressed through robust process control, regulatory oversight, and scientific innovation. Without these safeguards, scaling FWOFs risks shifting the burden from food waste reduction to soil and food system contamination.

### 2.4. Adoption of Food Waste–Based Organic Fertilizers

**Figure 3:**
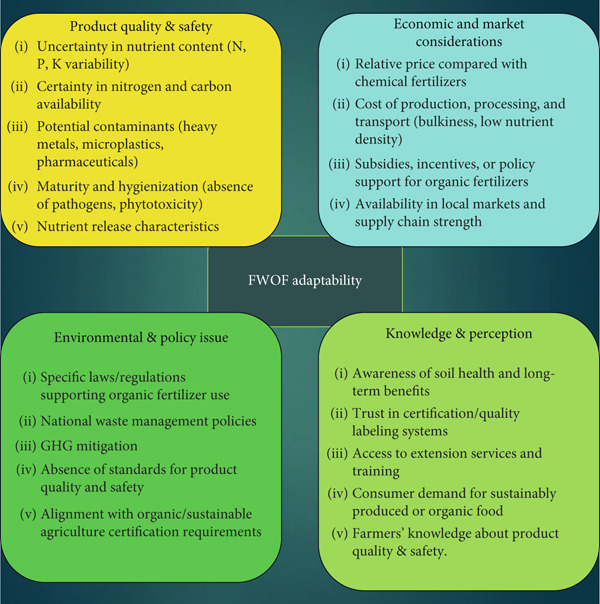
Factors may influence the adoption of FW‐based organic fertilizers.

The adoption of food waste–derived organic fertilizers (FWOFs) is shaped by an interplay of technical, economic, and social factors. Beyond simple availability, farmers’ willingness to adopt depends on many factors such as perceptions of product quality, cost competitiveness, ease of use, and the presence of supportive regulatory and extension frameworks (Figure 3). Field studies demonstrate that farmer education, access to extension services, and prior farming experience significantly increase the likelihood of FWOF adoption, largely because these attributes reduce uncertainty about nutrient content and product safety [31]. Also, adoption patterns may vary widely across regions, reflecting both technical and socioeconomic constraints. Economic variables, such as subsidies, access to credit, and proximity to markets, also play a decisive role: In Nigeria, adopters of organic fertilizers achieved higher household welfare, particularly among women and credit‐constrained farmers, highlighting the distributive dimension of adoption outcomes [32]. Conversely, barriers include bulkiness and transport costs, labor intensity of application, and inconsistent product quality, which deter many smallholders from shifting away from chemical fertilizers [33]. Moreover, the perceived agronomic effectiveness of FWOFs strongly influences adoption. Farmers are more likely to embrace these fertilizers when clear, locally relevant evidence demonstrates improvements in soil fertility, crop yield, or quality. However, heterogeneity in nutrient composition across food waste batches can generate unpredictable outcomes, undermining confidence and slowing adoption. Addressing this requires standardization protocols, quality certification, and simple nutrient labeling to reduce uncertainty. Social and cultural perceptions also play a crucial role. In some contexts, organic fertilizers are associated with traditional or low‐input farming, which may be perceived as less modern or less prestigious, limiting uptake among commercially oriented farmers. Conversely, increasing consumer demand for sustainably produced crops and the potential for organic certification can incentivize adoption, particularly in regions targeting export markets or premium domestic segments.

## 3. Conversion Method

### 3.1. Composting

Composting is one of the most widely applied methods for converting food waste into organic fertilizers due to its simplicity, low cost, and capacity to reduce waste volume while stabilizing nutrients. In aerobic composting, microorganisms degrade organic matter into a humified, nutrient‐rich product suitable for soil application. While its environmental and agronomic benefits are well‐documented, a critical evaluation shows that composting also faces limitations in nutrient conservation, process control, and scalability. Food waste compost provides organic matter that improves soil structure, water holding capacity, and microbial diversity [34]. Compost also releases nutrients more slowly than synthetic inputs, reducing leaching losses and improving long‐term soil fertility. Moreover, composting can inactivate many pathogens if thermophilic conditions (> 55°C) are maintained, producing a microbiologically safer product than raw waste. From a circular economy perspective, composting diverts biodegradable waste from landfills, reducing methane emissions and landfill burden [35].

Despite these advantages, composting is prone to nutrient losses, especially N. Ammonia volatilization during the thermophilic phase can result in N losses of 30%–50% if not carefully managed [36]. This compromises the agronomic value of the compost and increases air pollution. K is also prone to leaching during composting, particularly in high‐moisture FW. Furthermore, the final nutrient content is highly variable, depending on feedstock composition, C/N ratio, and process parameters. This variability creates challenges for standardization and market acceptance, as farmers cannot rely on a consistent nutrient profile. While composting reduces pathogens, incomplete process control may allow survival of *Salmonella* or *E. coli*, especially in decentralized or small‐scale operations [37]. Contamination with plastics and other inert materials is another concern, as many food waste streams contain packaging residues. Therefore, effective source separation and depackaging technologies are critical prerequisites for composting systems intended to supply safe, marketable fertilizer products.

Composting is relatively low cost compared to other valorization technologies such as AD or pyrolysis. However, its scalability is constrained by land availability, odor management, and time requirements. A typical composting cycle takes 6–12 weeks, which limits throughput and economic efficiency compared to faster conversion processes. In urban areas, odor and vector attraction pose additional social acceptance barriers [38]. Moreover, while large‐scale centralized facilities can achieve economies of scale, small‐scale operations are often less efficient and may struggle to meet regulatory safety standards.

### 3.2. Vermicomposting

Vermicomposting is a biological process in which earthworms, primarily *Eisenia fetida* and *Eudrilus eugeniae*, degrade organic waste into a nutrient‐rich biofertilizer known as vermicompost. Unlike conventional composting, which relies heavily on thermophilic microbial activity, vermicomposting is a mesophilic process where worms and associated microorganisms synergistically stabilize the substrate. Vermicompost is widely regarded as superior to traditional compost in terms of nutrient bioavailability and plant growth–promoting properties [39, 40]. However, vermicomposting faces significant constraints, particularly regarding feedstock suitability, pathogen control, and scalability [41]. Vermicompost is consistently reported to have higher nutrient availability compared to conventional compost, with improved concentrations of soluble N, P, and K. Earthworm activity enhances microbial diversity and produces humic substances that stimulate plant growth and soil structure improvement. Several studies have shown that vermicompost application not only improves crop yield but also enhances plant resistance to pests and diseases through induced systemic resistance (ISR) mechanisms [42]. These agronomic benefits give vermicompost an edge over many other organic fertilizers and provide strong justification for its adoption in sustainable farming systems.

Despite these benefits, vermicomposting is not universally applicable to all food waste streams. High‐fat, high‐protein, or oily wastes such as meat, dairy, or cooked food are unsuitable because they generate odors, attract pests, and may cause worm mortality. Optimal vermicomposting feedstocks are plant‐based residues such as vegetable trimmings, fruit peels, and cereal by‐products. This restricts the range of food wastes that can be valorized via vermicomposting and often necessitates pretreatment or blending with other wastes to achieve stable conditions. Thus, although nutrient‐rich, vermicompost production is limited by feedstock specificity and requires careful substrate management. Unlike thermophilic composting, vermicomposting does not achieve high temperatures that reliably inactivate pathogens. Earthworms can reduce microbial populations by digestion and microbial antagonism, but complete pathogen elimination is uncertain. Survival of *E. coli* and *Salmonella* has been reported in vermicomposted products when feedstock sanitation is inadequate [43]. Additionally, vermicomposting cannot remove heavy metals or microplastics, which may persist in the final product if present in the raw feedstock. This poses a regulatory challenge, as many countries require pathogen‐free, contaminant‐compliant fertilizers before market approval.

Vermicomposting is most successful at small to medium scales, particularly in decentralized systems such as household or community‐level waste management. Large‐scale industrial vermicomposting remains rare due to operational challenges: Maintaining optimal moisture (60%–80%), aeration, and pH conditions across large systems is difficult, and worm populations are highly sensitive to temperature fluctuations. Seasonal variations in tropical and temperate regions further constrain scalability. Moreover, while vermicompost commands a premium price in niche organic farming markets, its production cost is relatively high, limiting competitiveness with chemical fertilizers at scale.

### 3.3. AD

AD is increasingly promoted as a sustainable food waste valorization pathway, producing both renewable biogas and a nutrient‐rich by‐product known as digestate. Unlike composting and vermicomposting, which primarily generate soil amendments, this offers dual benefits: energy recovery and fertilizer production. This makes it an attractive option within the circular bioeconomy. However, while AD is technologically mature, the fertilizer potential of digestate remains underutilized and subject to critical challenges related to nutrient variability, contamination, and regulatory acceptance. Digestate is composed of solid and liquid fractions, both of which contain significant amounts of plant nutrients. The liquid fraction typically provides soluble N, primarily in the form of ammonium (NH₄^+^), which is immediately available to plants. In contrast, the solid fraction is richer in organic matter and P, supporting soil health and long‐term fertility. Unlike compost, AD preserves more N since anaerobic conditions minimize volatilization losses, often retaining 60%–80% of the initial N content. This makes digestate particularly valuable for reducing reliance on synthetic N fertilizers [44].

Despite its nutrient richness, digestate has limitations that constrain its widespread adoption. First, the nutrient composition is highly variable, depending on feedstock type, digester conditions, and posttreatment. FW‐based digestates are often unbalanced—rich in N but deficient in P and K, necessitating blending or supplementation to match crop needs. Second, the high NH₄^+^ content, while beneficial for plant uptake, can also cause phytotoxicity or N leaching if applied excessively or at the wrong time. Contamination is another critical concern. Digestates may retain pathogens if thermophilic digestion conditions are not met. Furthermore, heavy metals, microplastics, and pharmaceutical residues have been detected in FW‐derived digestates, raising regulatory and public health concerns. Unlike compost, AD does not eliminate such contaminants; instead, it often concentrates them in the solid fraction. These risks limit farmer acceptance and compliance with fertilizer quality standards in many regions [45].

AD systems are generally more capital‐intensive than composting or vermicomposting, requiring sophisticated infrastructure, monitoring, and skilled operation. While large‐scale AD plants are economically viable in Europe and China due to supportive policies and subsidies, their feasibility in low‐ and middle‐income countries is questionable. Digestate management is often considered a bottleneck: Without proper treatment or markets for digestate, the sustainability of AD systems is compromised. Land application remains the most common route, but logistical challenges reduce economic efficiency.

### 3.4. Biochar and Pyrolysis‐Based Fertilizers

Biochar production via pyrolysis represents a thermochemical approach to food waste valorization that differs fundamentally from biological pathways such as composting, vermicomposting, and AD. Pyrolysis involves heating organic matter at 300°C–700°C in the absence of oxygen, generating three outputs: biochar (solid), bio‐oil (liquid), and syngas (gaseous fuel). Among these, biochar has gained attention as a soil amendment and fertilizer carrier due to its stability, porosity, and nutrient retention properties. While widely promoted for its climate mitigation potential, biochar’s role as a direct fertilizer remains contested [46]. Biochar is better considered as a soil conditioner or nutrient delivery medium rather than a fertilizer substitute. Biochar improves soil physical and chemical properties by enhancing porosity, water retention, and cation‐exchange capacity. Its high surface area and functional groups make it an excellent carrier for nutrients, enabling the production of “engineered fertilizers” where biochar is enriched with N, P, or K during or after pyrolysis [47]. Such formulations have demonstrated improved nutrient use efficiency and reduced leaching losses compared to conventional fertilizers. In addition, biochar can immobilize toxic elements such as Cd and Pb, thereby reducing their bioavailability in soils. These properties make biochar especially relevant for degraded or contaminated soils.

Despite these benefits, biochar’s nutrient content is often low and highly variable. FW‐derived biochar tends to contain more K and P than N, since most N is volatilized during pyrolysis. As a result, biochar rarely meets crop nutrient demand on its own, requiring supplementation with other organic or mineral sources. Furthermore, biochar’s positive impacts on crop yield are frequently reported, but some studies show neutral or even negative effects. A 3‐year field trial in fertile soils found that biochar application did not increase crop yields and resulted in only minor and inconsistent changes in soil properties such as pH, electrical conductivity, and mineral N levels [48]. Similarly, a global meta‐analysis indicated that biochar application had no significant effect on crop yields in temperate regions, where soils are typically more fertile. In contrast, tropical soils, which are often more nutrient‐poor, showed a 25% average increase in crop yields with biochar application [49].

Another major uncertainty is the potential risk of contaminant concentration. Heavy metals present in food waste are not destroyed by pyrolysis; instead, they are concentrated in the biochar fraction. This raises safety concerns if food waste feedstocks include contaminated streams such as seafood waste (Hg and As) or municipal mixed waste (Pb and Cd). In addition, the production of polycyclic aromatic hydrocarbons (PAHs) during pyrolysis has been documented, which could pose environmental hazards if not properly managed [50]. From an economic perspective, pyrolysis requires higher capital investment and technical expertise compared to composting or vermicomposting. While biochar production is scalable, the technology is still underdeveloped for decentralized food waste management in low‐ and middle‐income countries. High energy requirements, limited markets for biochar‐based fertilizers, and a lack of regulatory frameworks hinder commercialization. However, coproduction of bioenergy (syngas and bio‐oil) can offset costs and improve feasibility in integrated biorefinery models.

## 4. Impact of FW‐Derived Organic Fertilizer

**Figure 4:**
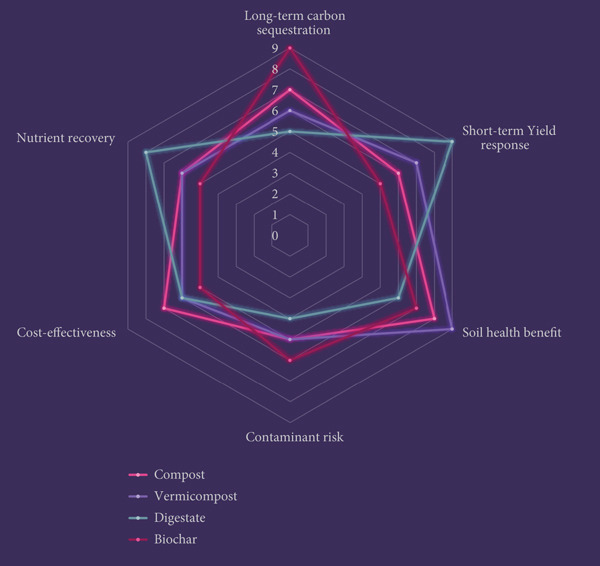
Comparative analysis of FWOF pathways, integrating agronomic performance and risk dimensions.

FWOFs exert multifaceted impacts that extend across soil systems, plant productivity, and the wider environment. Their application has been shown to improve soil organic matter, enhance microbial activity, and influence nutrient cycling, thereby contributing to long‐term soil health, yet they also introduce trade‐offs such as potential contaminant accumulation or nitrous oxide release under poor management (Figure 4). At the crop level, FWOFs can support yield stability, improve nutritional quality, and even stimulate plant defense responses, although results are sometimes inconsistent due to differences in feedstock composition, processing methods, and local agronomic conditions. From an environmental perspective, FWOFs offer clear benefits in reducing landfill disposal and greenhouse gas emissions. The overall impact of these fertilizers is therefore highly context‐dependent, requiring a critical evaluation of both their agronomic benefits and associated risks. Understanding these dynamics is essential to positioning FWOFs as reliable, sustainable alternatives to synthetic inputs within modern agriculture.

### 4.1. Soil Health

Compost and vermicompost add labile organic carbon that accelerates aggregation and microbial growth, improving early‐stage soil structure. Meta‐analytical and field evidence show increases in microbial biomass carbon and water‐stable aggregates after organic amendments, with particularly strong responses in the first years of use. Notably, a multisite comparison found that composted fields had substantially higher composite soil health scores than references, whereas solid digestate alone did not shift the average score, underscoring that not all organic fertilizers perform equally on soil health indices [51, 52]. Biochar contributes recalcitrant carbon that persists, elevates cation‐exchange capacity, and buffers acidity; however, the agronomic response depends on soil texture, baseline fertility, and climate. Recent reviews and meta‐analyses report improvements in water holding and microbial biomass, but with wide variance across contexts—highlighting that biochar functions best as a soil conditioner or carrier rather than a standalone fertilizer [53].

Vermicompost consistently stimulates microbial biomass and reshapes community composition via earthworm–microbe interactions, which can enhance plant–microbe symbioses relevant to nutrient cycling. A meta‐analysis concludes that earthworms are net mutualists of soil microorganisms, while experimental work shows that vermicompost increases microbial diversity and functional groups [54]. Complementarily, a quantitative synthesis reports that compost/vermicompost significantly improves legume nodulation and symbiotic N fixation, an explicitly biological soil health pathway [55]. Digestate effects on soil biota are mixed and strongly conditioned by its carbon quality and application mode. A critical review finds that many studies report neutral effects on microbial communities, with positive responses more likely when digestate contains higher organic C or is coapplied with carbonaceous materials (e.g., straw); this cautions against assuming biological benefits from nutrient‐rich but carbon‐poor liquid fractions [56].

Across FWOFs, improved aggregation and water retention are frequent but not universal. Compost additions commonly enhance aggregate stability and early‐season infiltration; in urban and compacted contexts, combining structural remediation with compost produced clear short‐term gains in physical and chemical soil health indicators. Biochar’s porous structure and surface functionality improve water holding capacity and can moderate acidity, benefits most evident in coarse‐textured or acidic soils [57, 58].

### 4.2. Nutrient Availability and Cycling

Organic fertilizer performance hinges on how quickly nutrients become plant available and how well soils retain them long enough to match crop demand. Across common products, N forms and P speciation differ markedly, so management must adapt to the material rather than treating “organic fertilizer” as a single category. Liquid digestate typically supplies fast‐acting NH₄^+^–N with little C to drive immobilization, so crops can take it up quickly, but losses via NH₃ volatilization or nitrification–leaching are a risk if surface‐applied ahead of rainfall. Field data show shallow‐disk injection reduces NH₃ loss by ~58% while conserving plant‐available N, underscoring that application method often matters more than product label for N recovery [59]. By contrast, composts release N slowly because much of their N is organically bound; recent work to predict mineralization from novel biobased fertilizers emphasizes the need for product‐specific coefficients rather than generic and highlights the large variance across feedstocks and processes [60]. A multiyear comparison of organic N sources further indicates that digestate and refined digestate products do not necessarily increase leaching relative to synthetic N when rates are aligned to crop demand, with extra N often retained via denitrification or soil organic pools—again pointing to management and dose as primary levers [61].

Meta‐analyses and lysimeter studies disagree on whether organic sources reduce or increase nitrate losses because outcomes pivot on soil texture, rainfall, and timing and whether the organic input is NH₄^+^‐rich (digestate) or C‐rich (compost). A global synthesis reports that organic inputs can lower N losses to water on average, yet other assessments find cases with equal or even higher leaching from organics [62, 63]. Coformulating or coapplying organics with sorbents (e.g., biochar) can further delay nitrate breakthrough and reduce soluble losses, although effects vary by biochar type and soil [64].

Digestate P is often dominated by inorganic orthophosphate and Mg/Ca phosphates. Its plant availability is generally high in the short term, but the fraction depends on feedstock and digester loading. Compost P contains more organic and Ca/Fe‐associated forms, releasing slowly via mineralization; long‐term reliance on compost alone can build soil test P and elevate loss risk in permeable urban or sandy soils [65]. Where P is scarce or runoff control is critical, recovered struvite (MgNH₄PO₄·6H₂O) offers a slow‐release alternative whose agronomic efficiency depends on soil pH and placement; meta‐analysis and recent field studies show positive yield and P uptake responses when struvite is localized near roots [66, 67]. Emerging work also shows P availability from digestate‐derived biochar scales with particle size and mineral phases—an engineering handle to tune release [68]. K in digestate is largely water‐soluble and rapidly available, which supports early crop demand but raises leaching concerns in coarse soils; multisite studies report increases in soil test K after digestate use, with magnitude controlled by product K content and application rate. Program design should therefore pair digestate with split applications or retention amendments on sands and monitor K alongside N and P [69].

### 4.3. Effect on Crop Production

FWOFs influence crop production not only through yield outcomes but also by enhancing crop quality and resilience. Field studies show that FWOFs enrich soil fertility and microbial activity while supplying nutrients that directly influence plant growth, yield, and resilience (Table 1). Furthermore, certain organic amendments stimulate systemic resistance in plants, reducing pest and disease incidence and lowering the need for chemical inputs. However, these benefits are inconsistent across studies, reflecting the influence of feedstock composition, application methods, soil conditions, and crop types. A critical assessment of these outcomes is therefore essential to determine when and where FWOFs deliver the greatest agronomic value.

**Table 1:** Effect of FWOFs on soil and plant growth.

**Food waste source**	**Fertilizer type**	**Crops**	**Major findings**	**Reference**
Food waste with *Sabah ragi*	Compost	Dwarf crape jasmine (*Tabernaemontana divaricata*)	Improved plant growth; increased germination index; increased pH value of growing medium; influenced water holding capacity and electrical conductivity.	[70]
Common food waste	Liquid organic fertilizer	Lettuce, cherry tomato, and cucumber	Higher nitrogen and phosphorus uptake in lettuce under hydroponic conditions, comparable to synthetic fertilizers, promotes sustainable nutrient recycling.	[71]
Mixed food waste	Anaerobic digestate	Leafy greens and some fruiting crops	Improved crop production’s sustainability; decreased emissions of greenhouse gas; enhanced plant growth; lessened plants’ vulnerability to salt stress.	[72]
Mixed food waste	Liquid digestate with fish sludge	*Lepidium sativum* and *Triticum aestivum*	Initially slowed germination and early growth; long‐term improvements with soil preincubation strategies.	[73]
Mixed food waste	Liquid digestate	Lettuce, eggplant, and *Brassica rapa*	Enhanced lettuce growth; decreased toxicity and nutrient leaching; increased soil biomass; no effect on soil pH; improved plant growth.	[74]
Food waste with biofertilizers	Dry form	Chinese cabbage seedlings	Increased abscisic acid content; increased root and leaf length and improved both dry and fresh weight; enhanced transpiration efficiency and chlorophyll content; increased mineral content; overall improved the plant growth.	[75]
Mixed food waste	Dehydrated form	*Zea mays* and *Triticum aestivum*	Influenced soil pH; improved soil electrical conductivity and microbial activity; higher application rates retarded the germination and decreased plant growth; lower application rates increased plant biomass and nutrient uptake.	[76]
Mixed fruit and vegetable waste	Biochar	Chickpea plants	Enhanced shoot and root biomass; elevated plant growth; improved capability for cation exchange and porosity of soil; increased water retention.	[77]
Mixed food waste	Biochar	Lettuce crop	Improved the quantum yield of Photosystem II; improved dehydrogenase activity; increased chlorophyll fluorescence content.	[78]
Mixed food waste	Anaerobic digestate	Tomato plants	Increased concentration of nitrogen in shoot and plant biomass; improved plant growth; reduced nitrogen mineralization.	[79]
Mixed food waste	Water extract and dry form	*Lactuca sativa* L., *Brassica rapa*, *Beta vulgaris* L., *Raphanus sativus* var. *niger*	Decreased biomass of naturally occurring weeds; improved plant growth under different conditions (warm and rainfall days); environmental conditions impacted effectiveness.	[80]
Common food waste	Mesophilic anaerobic digestate	Annual ryegrass	Increased both the root and shoot biomass; improved the growth of annual ryegrass; positive effect on the density of fungal hyphae.	[81]
Mixed food waste with rice husk	Biochar	Chinese cabbage	Significantly decreased heavy metal uptake and enhanced soil health; increased both quality and yield; biochar‐based mineral fertilizer positively affected the plant growth and use of nutrient efficiency.	[82]
Kitchen and table food waste	Anaerobic digestate	*Stachytarpheta jamaicensis* (L.) Vahl plant	Increased the chlorophyll content, height, and number of leaves; improved plant growth; enhanced microbial activity in soil.	[83]

#### 4.3.1. Yield Outcomes

Evidence from field trials and meta‐analyses shows that FW‐derived fertilizers can match mineral fertilizer yields in many systems, but the pattern of response differs by product and context. Compost or vermicompost tends to stabilize or gradually lift yields over multiple seasons. Digestate often gives an immediate yield bump if placed correctly, and rates are aligned to crop N demand. Biochar’s yield effect is the most variable and is strongest where soils are acidic, sandy, or degraded. Treating these materials as interchangeable “organics” obscures the real management rules that drive yield [84].

Long‐term comparisons show that compost is reliably associated with maintained or improved crop productivity through improved soil function, even when single season yield effects are modest. A recent cross‐site analysis found that compost (not solid digestate) raised average soil health scores and maintained crop yield, supporting the view that compost primarily boosts yield stability via soil structure and biology rather than a one‐off nutrient pulse [85]. Field evidence from dryland rotations likewise reports that higher compost rates frequently occupy the top yield ranks year after year, emphasizing stability under climatic variability [86, 87]. In vegetables, vermicompost repeatedly delivers sizable yield lifts: A 2‐year field study in peppers reported 28%–69% yield over farmer practice when vermicompost was integrated into the fertilization plan, and reviews focused on tomato production reach similar conclusions. These gains reflect both slow‐release N and stimulation of the rhizosphere, not just extra nutrients [88].

A meta‐analysis of 35 studies found that digestate increased yields by 80% versus unfertilized controls and produced yields statistically indistinguishable from mineral fertilization on average, though with high heterogeneity across sites and crops. Subgroup analyses showed parity with mineral N in wheat and maize, while barley lagged, highlighting the importance of crop species and timing [89]. A 4‐year field trial evaluated the effects of biogas digestate fertilization on winter wheat yield, N use efficiency, and N₂O emissions. Digestate application significantly boosted wheat yields (53%–83% higher than unfertilized controls), though first‐time treatments produced lower yields than long‐term applications [90]. Tactically, the application method is decisive. Shallow‐disk injection or banded placement reduces NH₃ loss by 40%–60% and preserves plant‐available N, translating to higher N recovery and more reliable yield responses than broadcast spreading. On maize, injected digestate has matched mineral N yields while cutting volatilization sharply, a finding echoed by broader syntheses of precision slurry/digestate placement [91]. At the same time, on‐farm trials show fraction matters: Liquid digestate can perform comparably to mineral N where variable rate application is used, while solid fractions may underperform on some sites without supplemental N. This cautions against assuming “digestate” is a single product class for yield planning [92].

Across hundreds of studies, biochar raises yield most reliably on coarse‐textured, acidic, or nutrient‐poor soils. On fertile temperate soils, multiyear field trials often detect little to no yield change despite clear gains in soil organic carbon and pH buffering. A recent 3‐year, seven‐biochar trial on two fertile soils reported no yield increase, even though soil properties shifted in favorable directions. This illustrates that biochar behaves more like a soil conditioner whose yield payoff emerges under constraint (drought, acidity, and low CEC) or when combined with nutrients [93]. A global dataset assembled in 2023 emphasizes the large variance in biochar yield effects across soils and climates, while newer studies in stressed systems (e.g., drought‐prone maize) do show significant yield gains when biochar improves water holding and nutrient retention. The signal is clear: expect biochar to raise yield stability in limiting environments, not universal yield jumps [94]. Couse with compost can shift the story. Recent work reports emergent yield benefits over several years with biochar plus compost coapplication, suggesting a route to combine short‐term biological stimulation (compost) with longer term conditioning (biochar) [95].

In cereals, yield response to FWOFs is governed by N timing and placement. Digestate can meet cereal N demand when injected or incorporated, matching mineral N in multiple field settings. Compost’s strength lies in buffering drought and maintaining yields across variable seasons, as noted in dryland wheat rotations and system‐level analyses. In vegetables (tomato, pepper, lettuce, etc.), vermicompost or stabilized compost frequently produces larger proportional yield increases than in cereals, likely because improvements in tilth, microbial activity, and micronutrient supply translate rapidly into marketable yield and quality [96].

#### 4.3.2. Crop Quality Improvements

Mechanisms linking FWOFs to improved crop quality include many factors. One of the important factors is enhanced nutrient bioavailability. FWOFs supply micronutrients and organic N in forms that promote balanced uptake and metabolic allocation to quality traits (e.g., vitamin and phenolic biosynthesis). Vermicompost and compost release micronutrients gradually and enhance root uptake via microbial‐mediated mobilization. Digestate supplies immediate N and soluble K, which can boost sugar accumulation and protein synthesis in grain [90]. Enhanced soil biological activity and plant–microbe interactions are another primary factor in improving the crop quality. Vermicompost particularly enriches beneficial microbes and humic substances that act as biostimulants, inducing phenolic pathways and improving fruit coloration and flavor compounds. Multiple studies link vermicompost amendments to higher microbial biomass and enzyme activities associated with nutrient mineralization, which correlate with quality metrics [97, 98]. Stress mitigation leads to concentration effects. By improving water holding (compost and biochar) or reducing salt stress, FW‐based organic fertilizer can concentrate solutes in plant tissues (raising soluble solids and antioxidants) and reduce nitrate accumulation. Under saline or drought stress, biochar and compost often increase antioxidant defenses and secondary metabolites [99].

Improving crop quality, such as nutrient density, functional compounds (antioxidants and polyphenols), and sensory attributes, is often the most compelling argument for using food waste–derived fertilizers. Several controlled and field studies show that FWOFs (especially vermicompost and well‐matured composts) increase micronutrient content (Fe, Zn, and Ca), improve fruit sugar/acid balance, and raise antioxidant and phenolic concentrations in vegetables and fruits. For example, vermicompost applications increased vitamin C and soluble solids and improved sugar:acid ratio in tomato under greenhouse conditions, while reducing nitrate accumulation compared with mineral fertilizer. These effects were linked to improved soil microbial activity and nutrient availability in the rhizosphere [100, 101].

Digestate (especially when tailored or fractionated) has been reported to increase dry matter, sugar contents, and firmness in greenhouse vegetables such as cucumber and tomato. Field wheat trials also report higher grain protein and sometimes higher micronutrient concentration after digestate application compared with unfertilized controls (and in some cases parity with mineral fertilizer) [90]. These short‐term quality gains reflect the high proportion of plant‐available N (NH₄^+^) and soluble K in many digestates. Biochar alone shows mixed results for quality metrics. In stressed environments (drought and saline soils), biochar increased fruit soluble solids and some antioxidant indices, likely by improving water availability and nutrient retention. However, on fertile soils, biochar rarely produced consistent quality improvements unless coapplied with compost or nutrient sources. Recent trials indicate that foliar nanobiochar or biochar plus compost blends may improve pigment concentration and secondary metabolites in tomato, but generalizability is limited, and mechanistic pathways remain partially resolved [102, 103].

#### 4.3.3. Pest and Disease Resistance

A study using Thai rice showed that vermicompost enriched with *Trichoderma asperelloides* triggered defense responses (e.g., increased defense‐related enzyme activities), improved seedling growth, and significantly reduced sheath blight incidence caused by *Rhizoctonia solani*, demonstrating ISR through beneficial microbial priming [104]. Another trial demonstrated that vermicompost fortified with *Trichoderma harzianum*, *Pseudomonas fluorescens*, and *Bacillus subtilis* notably suppressed *Fusarium* wilt in tomato. Treated plants had reduced disease severity, improved growth, and elevated antioxidant and defense enzyme activities compared to controls, highlighting ISR via microbial and biochemical pathways [105]. In an in vitro and seedling study, nonsterile compost and vermicompost (and their teas) inhibited *Rhizoctonia solani* (causing damping‐off in tomato seedlings) by 54%–67%, largely due to the dominance of *Trichoderma* spp. Notably, sterilizing compost abolished the suppression, indicating that microbial activity is essential for pathogen control [106]. A broader analysis found that composts made from recycled organic matter significantly reduced soil‐borne pathogen severity (pathogens across multiple crops) through both biotic (microbial competition) and abiotic (extractable C, NO₃–N, and pH) mechanisms [107].

Not all composts or vermicomposts confer disease suppression equally. Suppressiveness depends on the feedstock, composting method, microbial consortia, and maturity, which limits comparability and practical relevance. Most studies are greenhouse or controlled nursery assays; therefore, field‐scale validation is necessary, especially under diverse soil and pathogen pressure conditions. Compost teas may suppress pathogens but also risk human pathogen regrowth if nutrient management is not carefully controlled.

### 4.4. Inconsistent Outcomes Across Some Studies

Food waste is not a single “product.” Municipal kitchen waste, fruit/vegetable processing residues, bakery waste, and animal‐derived scraps differ in moisture, C:N, and N‐P‐K and contaminant loads. Studies and reviews consistently show that the nutrient content and contamination risk vary by feedstock and source region. So two studies that both call their amendment “food‐waste compost” may in reality be very different materials. This basic heterogeneity explains a large share of between‐study variance. How the waste is processed (composting vs. vermicomposting vs. AD vs. pyrolysis), and the exact process conditions, fundamentally alter nutrient form, stability, and pathogen/contaminant profiles. Immature composts are phytotoxic; liquid digestates supply mostly NH₄^+^ (fast‐acting) while composts supply organically bound N (slow release). Reviews of maturity indices and phytotoxicity demonstrate that failure to ensure maturity leads to contradictory plant responses between studies [108, 109].

Nutrient fractionation governs the timing of plant uptake. Compost often releases N slowly through mineralization (highly variable by feedstock and climate), whereas digestate provides mineral N that plants can use immediately but also lose rapidly if mismanaged. Meta‐analyses and incubation studies show large variation in mineralization rates across amendment types; this variance causes divergent yield outcomes [110, 111]. The same digestate broadcast on the surface will often give worse agronomic and environmental outcomes than the same material injected or banded (reduced NH₃ volatilization, higher N recovery, and better yields). Multiple field trials and mitigation studies demonstrate that placement and timing (synchrony with phenology and rainfall) explain many apparent contradictions. If a study used surface broadcast during wet weather, it is likelier to report lower N efficiency and poorer yield than one using injection in dry conditions. Thus, differences in application practice across experiments inflate heterogeneity [112].

Many promising reports come from greenhouse or pot trials (controlled and smaller scale) that amplify positive signals (better root environment and less weather variability). Long‐term, multisite field trials often show more moderate or context‐dependent effects because they integrate real variability. The predominance of short, small experiments in the literature, therefore, inflates apparent inconsistency when compared to field evidence [113]. Moreover, when products are contaminated or immature, negative effects on germination, seedling growth, or food safety can occur. These “bad batches” produce negative results that sit beside positive trials with clean, mature products. Studies of phytotoxicity and maturity underline this risk and call for routine testing [114].

## 5. Risks and Trade‐Offs

### 5.1. Contaminant Accumulation

Transitioning food waste into fertilizers closes nutrient loops, but it also shifts risks from waste streams into soils, crops, and the wider environment. Heavy metals (Cd, Pb, Cr, Ni, and Zn), microplastics, and organic contaminants have been repeatedly detected in composts, digestates, and food waste feedstocks. Systematic reviews and surveys show that heavy metal levels in many digestates and composts vary widely by feedstock and region; some products approach or exceed regulatory thresholds in sensitive markets, posing long‐term soil accumulation risks and food chain transfer concerns [115, 116]. Microplastics are now routinely reported in composts and digestates: Studies document fibers and fragments originating from packaging and tableware in municipal and processing waste, and microplastics are persistent in soils after amendment application. Reviews highlight that microplastics can alter soil physical properties and act as vectors for sorbed pollutants [117]. Pharmaceuticals and antibiotic residues occur in digestates and can alter soil microbial communities or select for antibiotic resistance determinants. Recent surveys show detectable levels of multiple pharmaceuticals in digestates across different countries, and experimental work links some compounds to changes in soil microbiome composition [118, 119].

Heavy metals are conserved or concentrated through biological and thermal processing, so repeated applications can lead to gradual soil enrichment and plant uptake. Microplastics survive biological treatment and are delivered to soil with amendments. Pharmaceuticals may persist through digestion or composting (depending on compound chemistry and process conditions) and exert sublethal effects on soil biota or accumulate in crops [120]. Future research should prioritize (1) source separation and depackaging to reduce plastics and associated contaminants at origin. (2) Implement routine multipollutant screening (heavy metals, a panel of pharmaceutically active compounds, and standardized MP metrics) for marketable batches. (3) Research on long‐term soil fate, crop uptake, and bioaccumulation of low‐level pharmaceuticals is urgent to inform safe application rates and withdrawal intervals.

### 5.2. Crop Safety Issues

Immature or insufficiently stabilized compost releases phytotoxic compounds (organic acids, ammonia, and high soluble salts) that reduce germination and damage seedlings. Seed germination and phytotoxicity index studies demonstrate that immature composts can severely depress establishment and early growth; this is a common cause when compost maturity tests are neglected in practice. Liquid digestate often contains high NH₄^+^ concentrations. Surface‐applied or overapplied digestates can emit ammonia (air pollution) and cause foliar or root phytotoxicity. They can increase short‐term soil salinity and pH, especially harmful for sensitive vegetables. Injection or incorporation, acidification, split applications, and rate control substantially reduce these hazards, but many operational contexts still rely on simple broadcast spreading [121].

Biochar has high sorptive capacity and can temporarily adsorb mineral N and labile P, reducing immediate plant availability (especially if uncharged or made from certain feedstocks/pyrolysis conditions). Several field studies report transient nutrient immobilization and null yield responses in the first seasons after biochar alone. These effects can be minimized by coapplication with compost/digestate or by precharging biochar with nutrients [122, 123]. Mandatory maturity testing for compost (e.g., germination index), specification and labeling for digestate (NH₄^+^ content, and solid fraction), and guidance for biochar conditioning (nutrient charging) are practical safeguards. Extension services must teach appropriate application methods (injection, incorporation, and split dosing) that transform risk into agronomic benefit.

### 5.3. Environmental Issue

Evidence on N₂O fluxes after application of FW‐based fertilizers is mixed and strongly conditional. Several controlled and field studies report N₂O peaks following digestate application, linked to high NH₄^+^ content, nitrification–denitrification pulses after rainfall or tillage, and soil moisture, while other studies find similar or lower N₂O emissions than mineral fertilizers when digestates are injected or stabilized. Meta‐analyses indicate that application method, timing, and soil conditions (texture and organic carbon) are dominant moderators of N₂O outcomes [124]. Redirecting food waste to composting, AD, or bioenergy pathways dramatically reduces landfill methane emissions, a major climate benefit. Life cycle analyses consistently show that recycling food waste through AD (with energy recovery) or well‐managed composting reduces overall greenhouse gas burdens compared with landfill disposal; provided transport and processing emissions are managed. Thus, the net climate trade‐off of FW‐to‐fertilizer schemes is usually positive, but the fertilizer application stage (N₂O risk) can erode some benefits if not managed [125].

Life cycle assessment (LCA) studies highlight that the climate and environmental performance of food waste valorization is pathway‐dependent. AD with energy recovery plus nutrient recycling often rates well. Composting fares better than landfill but may be outperformed by AD in energy terms. Pyrolysis/biochar can deliver long‐term carbon sequestration credits but requires careful accounting for energy inputs and potential contaminant concentration. Crucially, if digestate application leads to elevated N₂O or nitrate leaching, much of the GHG and water quality advantage can be lost, so integrated process‐to‐field management is essential.

## 6. Conclusion and Future Perspectives

The valorization of food waste into organic fertilizers offers a promising pathway to reduce environmental burdens, recycle nutrients, and enhance soil and crop productivity. Evidence from compost, vermicompost, digestate, and biochar applications demonstrates clear potential to improve soil organic matter, nutrient cycling, and crop quality while mitigating waste disposal challenges. However, adoption and outcomes remain inconsistent, reflecting variability in feedstock composition, processing methods, application practices, and local agroecological conditions. This inconsistency has hindered farmer confidence and slowed integration into mainstream agricultural systems. Critically, existing research on FWOFs remains fragmented, highly site‐specific, and often limited to short‐term trials, making it difficult to derive generalizable conclusions. Heterogeneity in feedstocks, lack of standardization in processing and product characterization, and incomplete monitoring of potential contaminants pose risks that cannot be overlooked.

Looking ahead, several priorities emerge. First, standardized product characterization (nutrient speciation, maturity, and contaminant testing) must be embedded in research and regulatory frameworks to ensure consistency and safety. Multiseason, multisite field trials are also urgently needed to capture context‐specific performance and environmental trade‐offs, moving beyond pot‐scale studies. In addition, the integration of LCAs will be essential to evaluate the net climate and environmental impacts across the collection, processing, transport, and field application stages. Economic analyses must assess cost–benefit trade‐offs under real‐world conditions, including labor and logistics, to inform policies and farmer decision‐making. Finally, policy instruments and social incentives, including subsidies, cooperative schemes, and education programs, should be designed to bridge the gap between scientific potential and farmer adoption. If these scientific, technical, and institutional challenges are addressed, FWOFs can transition from experimental inputs to reliable, certified, and widely adopted tools that simultaneously advance agricultural productivity, environmental sustainability, and circular economy goals.

## Conflicts of Interest

The authors declare no conflicts of interest.

## Funding

No funding was received for this manuscript.

## Data Availability

Data will be made available on request.
